# Exposures to multiple pesticides and the risk of Hodgkin lymphoma in Canadian men

**DOI:** 10.1007/s10552-013-0240-y

**Published:** 2013-06-12

**Authors:** Garthika Navaranjan, Karin Hohenadel, Aaron Blair, Paul A. Demers, John J. Spinelli, Punam Pahwa, John R. McLaughlin, James A. Dosman, Len Ritter, Shelley A. Harris

**Affiliations:** 1 Occupational Cancer Research Centre, 505 University Avenue, Toronto, ON M5G 1X3 Canada; 2 Dalla Lana School of Public Health, University of Toronto, Toronto, Canada; 3 Cancer Care Ontario, Toronto, Canada; 4 BC Cancer Agency, Vancouver, Canada; 5 School of Population and Public Health, University of British Columbia, Vancouver, Canada; 6 Department of Community Health and Epidemiology, University of Saskatchewan, Saskatoon, Canada; 7 Public Health Ontario, Toronto, Canada; 8 Canadian Centre for Health and Safety in Agriculture, University of Saskatchewan, Saskatoon, Canada; 9 University of Guelph, Guelph, Canada

**Keywords:** Hodgkin disease, Pesticides, Age, Case–control study, Population-based

## Abstract

**Purpose:**

To determine the risk of Hodgkin lymphoma (HL) associated with exposures to multiple pesticides grouped by various classes, including carcinogenic classifications.

**Methods:**

Data collected in the Cross-Canada Study of Pesticides and Health, a population-based incident case–control study in six provinces conducted between 1991 and 1994, were analyzed using unconditional logistic regression. Cases (*n* = 316) were identified through provincial cancer registries and hospital records. Controls (*n* = 1,506) were frequency-matched to cases by age (±2 years) within each province and were identified through provincial health records, telephone listings, or voter lists. The Cochran–Armitage test was used to check for trends within pesticide classes.

**Results:**

Overall, there was an increase in the risk of HL among all subjects who reported use of five or more insecticides (OR 1.88, 95 % CI 0.92–3.87) and among subjects younger than 40 who reported use of two acetylcholinesterase inhibitors (OR 3.16, 95 % CI 1.02–9.29). There was an elevated odds ratio associated with reported use of three or more probably carcinogenic pesticides (OR 2.47, 95 % CI 1.06–5.75), but no increase in risk for use of possibly carcinogenic pesticides. The risk of HL from reported use of fungicides or any pesticides was greater for cases diagnosed before age 40 than for cases diagnosed at or after age 40. When analyses excluded proxy respondents, OR estimates strengthened in some circumstances.

**Conclusions:**

This study found associations between HL and fungicides, insecticides, specifically acetylcholinesterase inhibitors, and pesticides previously identified as probable human carcinogens. These associations should be further evaluated, specifically in relation to age at diagnosis.

## Introduction

Hodgkin lymphoma (HL) is an important cancer with an estimated 67,000 new cases worldwide in 2008 [1]. HL has a bimodal age-incidence pattern with the highest rates seen in early adulthood (ages 15–40, especially in people in their 20s) and late adulthood (after 55) [2]. There are two main types of HL: (1) classic HL, which consists of four subtypes (nodular sclerosis, mixed cellularity, lymphocyte-rich, and lymphocyte-depleted), and (2) nodular lymphocyte predominance [2]. The causes of HL are poorly understood; however, some studies have suggested that certain agricultural practices might be associated with the development of HL [3]. Using data from the Cross-Canada Study of Pesticides and Health (CCSPH), Karunanayake et al. [4] and Pahwa et al. [5] previously found an elevated risk of HL from self-reported use of the insecticide chlorpyrifos [4] and the herbicide dichlorprop [5], respectively. A French case–control study found significant associations between HL and the use of fungicides, especially triazole fungicides, and urea herbicides [6]. Occupational exposure to phenoxyherbicides was associated with a statistically elevated risk of malignant lymphoma (HL and non-Hodgkin lymphoma) in a study from Sweden [7], but not in other studies [8, 9]. Finally, a study in the United States found that insecticide use on crops or animals was not associated with HL [10].

Most studies have examined the risk of HL from exposure to individual pesticides or broad groupings of pesticides. Few have focused on exposure to multiple pesticides or different combinations of pesticides routinely used together. This is important because these scenarios more accurately reflect how exposures occur in occupational or agricultural settings, and the evaluation of risks from exposures to chemical mixtures is important for human health risk assessment.

Hohenadel and colleagues used this approach to examine risks of non-Hodgkin lymphoma (NHL) from exposure to multiple pesticides and pesticide combinations using data from the CCSPH [11]. In this paper, we conduct a similar analysis for HL using data from the same study. Previous studies examining the risk from exposure to multiple pesticides have focused on NHL and have used broad groupings of pesticides such as insecticides, herbicides, and fungicides [11, 12]. These broad classes are comprised of many pesticides with varying chemical structures, which makes it difficult to identify individual pesticides that may increase risk or groups of pesticides with similar structure and/or function that may share a mechanism of carcinogenic action in humans. Examining pesticides by their mode of action in plants, insects, or fungi is another epidemiologic approach that can be used to identify chemical groups of concern. The objective of this work is to examine the risk of HL from lifetime exposures to multiple pesticides belonging to different classes and by mode of action. Because of the bimodal age incidence of HL, risks will also be examined by age group.

## Methods

### Study population

The data for this study on HL come from the CCSPH, a population-based incident case–control study conducted among male residents in six Canadian provinces (Ontario, Quebec, Alberta, British Columbia, Manitoba, and Saskatchewan). Men aged 19 years or older with a first diagnosis of soft-tissue sarcoma (STS), NHL, multiple myeloma (MM), and HL between 1 September 1991 and 31 December 1994 were included. Each province was given a target number of cases for each cancer site to balance the number of cases by province. The study size was determined based on a priori sample size calculations, and cases were ascertained from provincial cancer registries, except in Quebec where hospital ascertainment was used. Cancer registries and hospitals provided information to confirm the diagnosis, including pathology reports, although subjects for whom pathological material was unavailable remained in the study. Deceased individuals were not eligible as cases or controls, and surrogates for deceased individuals were not contacted. Proxy respondents were allowed for subjects eligible for the study. Subjects who were known to be HIV-positive were excluded. For controls, men aged 19 and older were selected at random from the provincial health insurance records (Alberta, Saskatchewan, Manitoba, Quebec), computerized telephone listings (Ontario), or voters’ lists (British Columbia). Controls were frequency-matched by age (±2 years) to cases within each province.

### Questionnaires

The postal and telephone interview questionnaires were modified versions of the telephone interview questionnaires used by studies focusing on pesticide exposure and cancer in Kansas and Nebraska [13, 14]. The postal questionnaire for the CCSPH was sent to all cases and controls to capture information on demographic characteristics, medical history, and other variables that could be potential confounders. Based on the results of a pilot study [15], a cumulative total exposure of 10 h per year to any pesticide combination was determined to be an appropriate cutoff for intensive exposure to pesticides. Both occupational and non-occupational (home, garden, hobby) uses of pesticides were considered in this classification of cumulative exposure. Participants who reported 10 or more hours of cumulative pesticide exposure per year on the postal questionnaire and a 15 % random sample of the participants who reported less than 10 h were sent a document listing pesticides and then interviewed by telephone to collect more detailed information about each pesticide used. The information collected on each pesticide included where the pesticide was used (work, home, or garden), the number of acres sprayed or treated if used on the farm, the year the pesticide was first used, how many years and days/year it was used, and how many of these days the pesticide was personally handled. The pesticides included in the questionnaire were those that had ever been registered for use in Canada and reviewed by IARC; pesticides that were recently banned or restricted in Canada; and pesticides commonly used in Canada for specific purposes. A more detailed discussion of the recruitment procedure, study design, and data collection has been published elsewhere [16].

### Statistical analyses

#### Exposure to multiple pesticides

We examined the risk of HL by the total number of pesticides ever used (0, 1, 2–4, and 5+ pesticides). Similar analyses were conducted for the number of pesticides used grouped by class, that is, herbicides, insecticides, and fungicides. Analyses were also conducted to examine the number of phenoxyherbicides used to further investigate the mixed findings of other studies and urea herbicides to confirm the findings of other studies.

The risk of HL was also evaluated according to the number of possibly carcinogenic pesticides used. These were pesticides considered possibly carcinogenic or higher by the International Agency for Research on Cancer (IARC) [17], suggestive evidence of carcinogenic potential or higher (2005), known/likely to be carcinogenic (1996), or possible human carcinogen or higher (1986 classification) by the United States Environmental Protection Agency (EPA) Office of Pesticides Program (OPP), or EPA Integrated Risk Information System (IRIS) [18, 19]. A more stringent level of carcinogenic pesticides (probably carcinogenic) was also used to evaluate and compare the risk of HL. A pesticide was considered probably carcinogenic if it was classified as probably carcinogenic to humans or higher by IARC [17], or likely to be carcinogenic to humans or higher (2005 classification), known/likely to be carcinogenic (1996 classification), or probable human carcinogen or higher (1986 classification) by EPA OPP, or the EPA IRIS [18, 19]. The list of pesticides classified as possibly and probably carcinogenic used in the analysis is presented in “Appendix 1”.

The risk of HL was also examined by the number of pesticides used with a similar mode of action in target species. These groups of pesticides were chosen for investigation if there was an adequate number of exposed individuals and there was more than one pesticide belonging to that group. Based on these criteria, the following modes of pesticidal action presented by Costa [20] were used for insecticides: (A) acetylcholinesterase inhibitor; (B) sodium channel activator; (C) GABA receptors-gated chloride channel inhibitor and for herbicides; and (D) auxin growth regulator (please refer to “Appendix 2” for the pesticides found in each group). Fungicides could not be grouped according to common modes of action due to small numbers.

These analyses have been restricted to the self-reported “ever/never” use of individual pesticides and various pesticide groupings. Since use and subsequent exposure to pesticides in the home and garden on a seasonal basis may be different from occupational settings, we repeated the above analyses evaluating home use and work use separately.

The risk of HL from reported use of various pesticides was evaluated using unconditional logistic regression. Interactions between age and the pesticide variables were also tested in each model for class, carcinogenicity, mode of action because of the bimodal age-incidence pattern for HL suggesting the possibility of two different etiologies. Those models with significant interaction terms were then stratified and analyzed by subjects younger than 40 and older than or equal to 40. Models for the multiple pesticides were adjusted for age, as a continuous variable, and province since controls were age-frequency-matched to cases in each province. Variables that might be potential confounders of the relationship between multiple pesticide exposure and the risk of HL were investigated. Based on a review of the literature, possible a priori confounders included ethnicity, diagnosis of cancer in an immediate family member, smoking, level of education, and use of a proxy respondent. The variable for ethnicity used in this study included the following categories: North American, Scandinavian, Eastern European, Western European, Asian, British, and other. The variable used for smoking was a binary variable for having ever smoked. Individuals with missing data for a variable of interest were placed into a “missing” category for that variable and were included in all models. Confounders that changed the odds ratios by at least 10 % were included in the final multivariate model. Trends for the number of pesticides used within each pesticide class were examined using the Cochran-Armitage exact trend test. All analyses were conducted using SAS version 9.2 (SAS Institute Inc.).

The University of Toronto Health Sciences Research Ethics Board reviewed and approved the protocol for these analyses.

## Results

### Study population

Table 1 characterizes the study population. This dataset contained information on 316 HL cases (68.4 % of those contacted) and 1,506 controls (48 % of those contacted). There was no information collected on non-respondents to assess reasons for non-participation. The largest number of cases and controls was from Ontario (39 %). Controls were frequency-matched to cases of HL, NHL, MM, and STS resulting in an average younger age for HL cases than controls because of the bimodal age-incidence distribution for HL. Compared to cases, a slightly higher proportion of controls required a proxy respondent, likely due to the older age of the control group. Sensitivity analyses were also performed excluding subjects whose information was reported by a proxy respondent or whose respondent status was missing to evaluate potential bias.

**Table 1:** Characteristics of Hodgkin lymphoma cases and controls in the study population

	Cases (*n* = 316)		Controls (*n* = 1,506)		*p* value
	Mean	SD	Mean	SD	
Age	40.09	15.91	54.08	16.35	<0.0001
	*n*	**%**	*n*	**%**	
Province					
Alberta	54	17.09	196	13.01	
Saskatchewan	16	5.06	91	6.04	
Manitoba	21	6.65	113	7.50	
Ontario	122	38.61	585	38.84	
Quebec	52	16.46	291	19.32	
British Columbia	51	16.14	230	15.27	0.40
Respondent					
Subject	279	88.29	1,286	85.39	
Proxy	26	8.23	153	10.16	
Missing	11	3.48	67	4.45	0.40
Immediate family diagnosed with cancer					
Yes	104	32.91	498	33.07	
No	202	63.92	973	64.61	
Unknown/missing	10	3.16	35	2.32	0.68
Ever smoked					
Yes	186	58.86	970	64.45	
No	128	40.51	526	34.95	
Unknown/missing	2	0.63	10	0.66	0.17
Education					
Not completed high school	71	22.47	510	33.86	
Completed high school	58	18.35	213	14.14	
More than high school	134	42.41	518	34.40	
Completed university	51	16.14	247	16.40	
Unknown	2	0.63	18	1.20	0.0007

### Multiple pesticides

#### Pesticides by class and carcinogenicity

Table 2 presents the results from analyses examining pesticide class and possible or probable carcinogenicity, and age at diagnosis. Table 3 presents results excluding proxy respondents. The interaction term for age was borderline significant for the any pesticides (*p* = 0.06) and fungicides (*p* = 0.05) classes at the 5 % level of significance (data not shown). Therefore, results for these classes are presented in Tables 2 and 3 stratified by age. The risk of HL was elevated and showed evidence of a positive trend by number of pesticides used for the younger age group, but not in the older age group (ORs for five or more pesticides were 1.79 (95 % CI 0.93–3.43) for <40 years and 0.84 (95 % CI 0.46–1.54) for >40 years) and for fungicides (ORs for two or more fungicides were 3.76 (95 % CI 0.96–14.75) for <40 years and 0.19 (95 % CI 0.03–1.39) for >40 years). Although examination of other classes of pesticides did not show significant interaction with age, there was a nearly twofold increase in the risk of HL among individuals who reported use of five or more insecticides (OR 1.88, 95 % CI 0.92–3.87), and this association was stronger when proxy respondents were excluded (OR 2.20, 95 % CI 1.02–4.74). Reported use of any herbicide, any phenoxyherbicide, or any urea herbicide was not associated with the risk of HL. The largest odds ratio was observed for exposure to three or more probably carcinogenic pesticides (OR 2.47, 95 % CI 1.06–5.75), although the trend was not significant. No effect was seen for exposure to three or more possibly carcinogenic pesticides (OR 1.18, 95 % CI 0.63–2.21) (Table 2). For trends by number of pesticides used, an increasing trend was of borderline significance for fungicides among those <40 years (*p* = 0.06) and significant for an inverse trend among those >40 years (*p* = 0.05). No other trends were statistically significant.

**Table 2:** Effect of multiple pesticide exposure on the risk of HL by class and carcinogenicity

Number of pesticides used	Total cases *n* (%)	Total controls *n* (%)	Overall OR^a,b^ (95 % CI)	Work-related OR^a,b^ (95 % CI)	Home-related OR^a,b^ (95 % CI)
Any pesticides-subjects <40 years old					
0	129 (70.49)	261 (73.31)	1.00	1.00	1.00
1	8 (4.37)	21 (5.90)	0.98 (0.41–2.31)	0.55 (0.17–1.73)	0.93 (0.42–2.06)
2–4	24 (13.11)	47 (13.20)	1.12 (0.64–1.95)	1.46 (0.60–3.55)	1.19 (0.67–2.11)
5+	22 (12.02)	27 (7.58)	1.79 (0.93–3.43)	1.80 (0.82–3.93)	1.89 (0.64–5.60)
			*p* (trend) = 0.12	*p* (trend) = 0.08	*p* (trend) = 0.23
Any pesticides-subjects >40 years old					
0	99 (74.44)	836 (72.70)	1.00	1.00	1.00
1	7 (5.26)	39 (3.39)	1.41 (0.60–3.31)	0.45 (0.10–1.92)	1.22 (0.62–2.42)
2–4	12 (9.02)	128 (11.13)	0.75 (0.39–1.42)	0.64 (0.22–1.81)	0.84 (0.45–1.57)
5+	15 (11.28)	147 (12.78)	0.84 (0.46–1.54)	1.02 (0.43–2.41)	0.68 (0.28–1.62)
			*p* (trend) = 0.26	*p* (trend) = 0.26	*p* (trend) = 0.31
Any fungicide-subjects <40 years old					
0	165 (90.16)	334 (93.82)	1.00	1.00	1.00
1	13 (7.10)	18 (5.06)	1.32 (0.60–2.89)	1.61 (0.68–3.83)	0.48 (0.06–4.19)
2–4	5 (2.73)	4 (1.12)	3.76 (0.96–14.75)	**4.72 (1.08**–**20.6)**	–
			*p* (trend) = 0.06	***p*** **(trend)** **=** **0.02**	
Any fungicide-subjects >40 years old					
0	123 (92.48)	1,027 (89.30)	1.00	1.00	1.00
1	9 (6.77)	72 (6.26)	0.96 (0.46–1.99)	0.92 (0.35–2.43)	0.72 (0.21–2.43)
2–4	1 (0.75)	51 (4.43)	0.19 (0.03–1.39)	0.23 (0.03–1.73)	–
			*p* (trend) = 0.05	*p* (trend) = 0.06	
Any insecticide					
0	238 (75.32)	1,154 (76.63)	1.00	1.00	1.00
1	33 (10.44)	127 (8.43)	1.16 (0.75–1.79)	0.76 (0.35–1.62)	1.24 (0.81–1.90)
2–4	33 (10.44)	189 (12.55)	0.92 (0.60–1.40)	**1.98 (1.10**–**3.56)**	0.86 (0.54–1.38)
5+	12 (3.80)	36 (2.39)	1.88 (0.92–3.87)	2.27 (0.54–9.61)	0.91 (0.20–4.15)
			*p* (trend) = 0.35	***p*** **(trend)** **=** **0.02**	*p* (trend) = 0.30
Any herbicide					
0	238 (75.32)	1,148 (76.23)	1.00	1.00	1.00
1	30 (9.49)	146 (9.69)	1.06 (0.69–1.65)	0.72 (0.35–1.49)	1.07 (0.69–1.65)
2–4	37 (11.71)	151 (10.03)	1.17 (0.77–1.79)	1.38 (0.72–2.65)	1.08 (0.65–1.79)
5+	11 (3.48)	61 (4.05)	0.77 (0.38–1.55)	0.98 (0.48–2.01)	–
			*p* (trend) = 0.40	*p* (trend) = 0.32	*p* (trend) = 0.27
Any phenoxy herbicide					
0	252 (79.75)	1,188 (78.88)	1.00	1.00	1.00
1	36 (11.39)	183 (12.15)	0.94 (0.63–1.41)	0.67 (0.29–1.55)	0.97 (0.63–1.49)
2	18 (5.70)	86 (5.71)	1.01 (0.57–1.78)	0.88 (0.34–2.27)	1.07 (0.52–2.17)
3+	10 (3.16)	49 (3.25)	1.01 (0.48–2.11)	1.09 (0.50–2.40)	2.90 (0.27–31)
			*p* (trend) = 0.43	*p* (trend) = 0.38	*p* (trend) = 0.43
Urea herbicides					
0	309 (97.8)	1,490 (98.9)	1.00	1.00	1.00
1+	7 (2.2)	16 (1.1)	1.63 (0.63–4.19)	1.75 (0.63–4.82)	–
Possibly carcinogenic pesticides and higher					
0	246 (77.85)	1,162 (77.16)	1.00	1.00	1.00
1	23 (7.28)	107 (7.10)	0.90 (0.54–1.48)	0.75 (0.34–1.69)	0.88 (0.55–1.41)
2–4	32 (10.13)	171 (11.35)	0.96 (0.62–1.48)	0.86 (0.44–1.70)	1.08 (0.67–1.75)
5+	15 (4.75)	66 (4.38)	1.18 (0.63–2.21)	1.62 (0.78–3.36)	1.18 (0.26–5.41)
			*p* (trend) = 0.43	*p* (trend) = 0.37	*p* (trend) = 0.43
Probably carcinogenic pesticides and higher					
0	281 (88.92)	1,317 (87.45)	1.00	1.00	1.00
1	17 (5.38)	112 (7.44)	0.75 (0.43–1.30)	0.73 (0.33–1.60)	0.84 (0.48–1.47)
2	9 (2.85)	54 (3.59)	1.17 (0.54–2.52)	**3.36 (1.33**–**8.52)**	1.24 (0.46–3.36)
3+	9 (2.85)	23 (1.53)	**2.47 (1.06**–**5.75)**	3.02 (0.69–13)	–
			*p* (trend) = 0.46	*p* (trend) = 0.09	*p* (trend) = 0.21

^a^ORs adjusted for age and province of residence

^b^ The bold signifies those odds ratios or trends that are statistically significant

**Table 3:** Effect of multiple pesticide exposure on the risk of HL by class and carcinogenicity, excluding proxy respondents

Number of pesticides used	Total cases *n* (%)	Total controls *n* (%)	Overall OR^a,b^ (95 % CI)	Work-related OR^a,b^ (95 % CI)	Home-related OR^a,b^ (95 % CI)
Any pesticides-subjects <40 years old					
0	114 (68.7)	225 (72.6)	1.00	1.00	1.00
1	8 (4.82)	19 (6.13)	1.05 (0.44–2.52)	0.57 (0.18–1.82)	0.98 (0.44–2.21)
2–4	23 (13.9)	41 (13.23)	1.13 (0.63–2.03)	1.45 (0.59–3.53)	1.23 (0.67–2.24)
5+	21 (12.7)	25 (8.06)	1.75 (0.89–3.44)	1.67 (0.74–3.77)	2.08 (0.68–6.39)
			*p* (trend) = 0.09	*p* (trend) = 0.10	*p* (trend) = 0.14
Any pesticides-subjects >40 years old					
0	80 (70.8)	700 (71.2)	1.00	1.00	1.00
1	7 (6.2)	35 (3.59)	1.63 (0.68–3.90)	0.50 (0.12–2.17)	1.35 (0.66–2.79)
2–4	11 (9.7)	113 (11.58)	0.83 (0.42–1.64)	0.64 (0.19–2.13)	1.02 (0.54–1.94)
5+	15 (13.3)	128 (13.11)	1.04 (0.56–1.94)	1.21 (0.49–2.95)	0.82 (0.34–2.00)
			*p* (trend) = 0.50	*p* (trend) = 0.40	*p* (trend) = 0.50
Any fungicide-subjects <40 years old					
0	149 (89.8)	289 (93.2)	1.00	1.00	1.00
1	12 (7.23)	17 (5.48)	1.20 (0.53–2.69)	1.47 (0.59–3.63)	0.46 (0.052–3.98)
2–4	5 (3.01)	4 (1.29)	3.45 (0.88–13.5)	4.33 (0.99–18.97)	–
			*p* (trend) = 0.09	***p*** **(trend)** **=** **0.03**	
Any fungicide-subjects >40 years old					
0	104 (92.0)	871 (89.2)	1.00	1.00	1.00
1	8 (7.1)	57 (5.84)	1.07 (0.49–2.34)	0.93 (0.31–2.77)	0.83 (0.24–2.84)
2–4	1 (0.88)	48 (4.92)	0.21 (0.03–1.53)	0.25 (0.03–1.89)	–
			*p* (trend) = 0.08	***p*** **(trend)** **=** **0.03**	
Any insecticide					
0	204 (73.12)	977 (75.97)	1.00	1.00	1.00
1	31 (11.11)	108 (8.40)	1.30 (0.82–2.07)	0.90 (0.41–1.97)	1.43 (0.91–2.25)
2–4	33 (11.83)	170 (13.22)	1.02 (0.66–1.58)	**2.19 (1.19**–**4.02)**	0.95 (0.58–1.54)
5+	11 (3.94)	31 (2.41)	**2.20 (1.02**–**4.74)**	1.81 (0.33–9.79)	1.29 (0.28–5.93)
			*p* (trend) = 0.21	***p*** **(trend)** **=** **0.01**	*p* (trend) = 0.47
Any herbicide					
0	204 (73.12)	970 (75.43)	1.00	1.00	1.00
1	28 (10.04)	128 (9.95)	1.17 (0.74–1.85)	0.70 (0.33–1.51)	1.23 (0.77–1.94)
2–4	36 (12.90)	136 (10.58)	1.25 (0.81–1.95)	1.40 (0.70–2.81)	1.23 (0.73–2.08)
5+	11 (3.94)	52 (4.04)	0.91 (0.44–1.87)	1.11 (0.53–2.33)	–
			*p* (trend) = 0.22	*p* (trend) = 0.23	*p* (trend) = 0.16
Any phenoxy herbicide					
0	218 (78.14)	1,006 (78.23)	1.00	1.00	1.00
1	33 (11.83)	160 (12.44)	0.99 (0.64–1.52)	0.54 (0.20–1.42)	1.11 (0.71–1.74)
2	18 (6.45)	76 (5.91)	1.14 (0.63–2.05)	0.98 (0.37–2.59)	1.16 (0.56–2.41)
3+	10 (3.58)	44 (3.42)	1.13 (0.53–2.41)	1.24 (0.55–2.79)	3.73 (0.30–46.04)
			*p* (trend) = 0.43	*p* (trend) = 0.48	*p* (trend) = 0.29
Urea herbicides					
0	272 (97.5)	1,271 (98.8)	1.00	1.00	1.00
1+	7 (2.51)	15 (1.17)	1.78 (0.68–4.66)	1.77 (0.63–4.91)	1.78 (0.11–29.20)
Possibly carcinogenic pesticides and higher					
0	212 (76.0)	984 (76.52)	1.00	1.00	1.00
1	22 (7.89)	92 (7.15)	0.97 (0.57–1.63)	0.73 (0.31–1.74)	1.02 (0.63–1.67)
2–4	31 (11.11)	150 (11.66)	1.06 (0.67–1.67)	0.97 (0.48–1.96)	1.19 (0.72–1.98)
5+	14 (5.02)	60 (4.67)	1.26 (0.65–2.42)	1.70 (0.79–3.69)	1.29 (0.28–6.01)
			*p* (trend) = 0.46	*p* (trend) = 0.28	*p* (trend) = 0.46
Probably carcinogenic pesticides and higher					
0	246 (88.17)	1,118 (86.94)	1.00	1.00	1.00
1	16 (5.73)	102 (7.93)	0.77 (0.43–1.37)	0.77 (0.35–1.71)	0.88 (0.49–1.57)
2	9 (3.23)	44 (3.42)	1.53 (0.69–3.37)	**4.96 (1.80**–**13.65)**	1.55 (0.57–4.24)
3+	8 (2.87)	22 (1.71)	2.42 (0.99–5.95)	2.61 (0.45–14.99)	–
			*p* (trend) = 0.42	*p* (trend) = 0.08	*p* (trend) = 0.16

^a^ ORs adjusted for age and province of residence

^b^ The bold signifies those odds ratios or trends that are statistically significant

The analyses evaluating work versus home exposures (Table 2) tended to show that trends were stronger and ORs larger when only work use was considered (specifically, any fungicide use in men under 40 years, insecticide use, and probably carcinogenic pesticides). ORs for phenoxy herbicide use, however, were slightly larger for home use.

Overall, the analyses excluding proxy respondents had ORs in the same direction, and for most exposure groups, the ORs were slightly higher. The only exception was the fungicides group for the younger than 40 age group, which had lower ORs.

Odds ratios did not change significantly when adjusting for a priori confounders including ethnicity, smoking, level of education, diagnosis of cancer in an immediate family member (results not presented), and thus, these were not controlled for in any of the analyses involving multiple pesticides.

#### Pesticides by mode of action

Table 4 presents odds ratios for HL by the number of pesticides reportedly used grouped by their pesticidal mode of action. Table 5 presents these results excluding proxy respondents. The interaction term for age was borderline significant for use of acetylcholinesterase inhibitors (*p* = 0.07) (data not shown). Therefore, results for these classes are presented in Tables 4 and 5 stratified by age. The risk of HL was elevated and showed evidence of a positive trend by number of acetylcholinesterase inhibitors used for the younger age group, but not in the older age group (ORs for two pesticides were 3.16 (95 % CI 1.02–9.29) for <40 years and 0.49 (95 % CI 0.15–1.62) for >40 years). The association for the younger age group became slightly stronger after excluding information provided by proxy respondents (OR for two pesticides was 3.69, 95 % CI 1.12–12.15). For GABA receptors-gated chloride channel inhibitors, the OR was increased for use of two or more of such pesticides (OR 2.45, 95 % CI 0.47–12.92), but there were only two exposed cases and the increase was not statistically significant. Pesticides regulating auxin growth did not appear to increase the risk of HL. When the pesticides evaluated by mode of action were stratified by home versus work use, ORs were greater in magnitude for work-related exposures, in particular for the acetylcholinesterase inhibitors (using three or more in the younger than 40 age group OR 11.15, 95 % CI 1.15–108.2). When excluding proxy respondents from analyses by mode of action, the direction of effect remained the same, and for most exposure groups, the magnitude of the odds ratios increased.

**Table 4:** Effect of multiple pesticide exposure on the risk of HL by pesticide mode of action

Pesticide mode of action	Total cases *n* (%)	Total controls *n* (%)	Overall OR^a,b^ (95 % CI)	Work-related OR^a,b^ (95 % CI)	Home-related OR^a,b^ (95 % CI)
Acetylcholinesterase inhibitors <40 years old					
0	156 (85.25)	324 (91.01)	1.00	1.00	1.00
1	14 (7.65)	22 (6.18)	1.40 (0.67–2.94)	1.38 (0.49–3.87)	1.96 (0.87–4.43)
2	8 (4.37)	6 (1.69)	**3.16 (1.02**–**9.29)**	3.49 (0.73–16.69)	2.71 (0.58–12.58)
3+	5 (2.73)	4 (1.12)	3.92 (0.91–16.96)	**11.15 (1.15**–**108.2)**	**–**
			***p*** **(trend)** **=** **0.01**	***p*** **(trend)** **=** **0.008**	*p* (trend) = 0.12
Acetylcholinesterase inhibitors >40 years old					
0	275 (87.03)	1,317 (87.45)	1.00	1.00	1.00
1	20 (6.33)	108 (7.17)	0.53 (0.22–1.25)	0.73 (0.21–2.55)	0.65 (0.27–1.56)
2	11 (3.48)	57 (3.78)	0.49 (0.15–1.62)	1.79 (0.37–8.75)	0.45 (0.11–1.91)
3+	10 (3.16)	24 (1.59)	2.02 (0.72–5.67)	5.97 (0.56–64.0)	1.19 (0.26–5.51)
			*p* (trend) = 0.45	*p* (trend) = 0.23	*p* (trend) = 0.22
Sodium channel activators					
0	306 (96.84)	1,442 (95.75)	1.00	1.00	1.00
1	10 (3.16)	64 (4.25)	1.08 (0.53–2.19)	1.58 (0.61–4.07)	0.87 (0.33–2.30)
GABA receptors-gated chloride channels inhibitors					
0	292 (92.41)	1,382 (91.77)	1.00	1.00	1.00
1	22 (6.96)	117 (7.77)	0.98 (0.59–1.62)	1.80 (0.65–5.0)	0.83 (0.47–1.46)
2+	2 (0.63)	7 (0.46)	2.45 (0.47–12.92)	3.12 (0.54–18.03)	–
			*p* (trend) = 0.45	*p* (trend) = 0.17	
Auxin growth regulator					
0	252 (79.75)	1,187 (78.82)	1.00	1.00	1.00
1	34 (10.76)	177 (11.75)	0.91 (0.60–1.38)	0.79 (0.36–1.75)	0.95 (0.62–1.46)
2–4	24 (7.59)	123 (8.17)	0.99 (0.60–1.62)	0.75 (0.35–1.61)	1.19 (0.61–2.32)
5+	6 (1.90)	19 (1.26)	1.31 (0.49–3.52)	1.65 (0.60–4.57)	–
			*p* (trend) = 0.50	*p* (trend) = 0.45	*p* (trend) = 0.49

^a^ORs adjusted for age and province of residence

^b^ The bold signifies those odds ratios or trends that are statistically significant

**Table 5:** Effect of multiple pesticide exposure on the risk of HL by pesticide mode of action, excluding proxy respondents

Pesticide mode of action	Total cases *n* (%)	Total controls *n* (%)	Overall OR^a,b^ (95 % CI)	Work-related OR^a,b^ (95 % CI)	Home-related OR^a,b^ (95 % CI)
Acetylcholinesterase inhibitors <40 years old					
0	140 (84.34)	282 (90.97)	1.00	1.00	1.00
1	14 (8.43)	19 (6.13)	1.46 (0.68–3.14)	1.48 (0.52–4.23)	2.08 (0.90–4.83)
2	8 (4.82)	5 (1.61)	**3.69 (1.12**–**12.15)**	3.33 (0.70–15.91)	4.41 (0.79–24.50)
3+	4 (2.41)	4 (1.29)	3.18 (0.65–15.57)	8.89 (0.81–97.33)	–
			***p*** **(trend)** **=** **0.02**	***p*** **(trend)** **=** **0.02**	*p* (trend) = 0.08
Acetylcholinesterase inhibitors >40 years old					
0	99 (87.61)	840 (86.07)	1.00	1.00	1.00
1	6 (5.31)	72 (7.38)	0.63 (0.26–1.53)	0.96 (0.27–3.46)	0.73 (0.30–1.78)
2	3 (2.65)	47 (4.82)	0.55 (0.17–1.85)	1.98 (0.39–10.00)	0.51 (0.12–2.20)
3+	5 (4.42)	17 (1.74)	2.56 (0.88–7.46)	7.23 (0.65–80.34)	1.53 (0.32–7.30)
			*p* (trend) = 0.41	*p* (trend) = 0.15	*p* (trend) = 0.35
Sodium channel activators					
0	270 (96.8)	1,228 (95.49)	1.00	1.00	1.00
1	9 (3.23)	58 (4.51)	1.10 (0.52–2.35)	1.45 (0.52–4.09)	1.02 (0.38–2.73)
GABA receptors-gated chloride channels inhibitors					
0	257 (92.11)	1,175 (91.37)	1.00	1.00	1.00
1	20 (7.17)	105 (8.16)	0.98 (0.58–1.67)	1.75 (0.56–5.49)	0.86 (0.47–1.56)
2+	2 (0.72)	6 (0.47)	2.88 (0.52–15.81)	3.30 (0.56–19.33)	–
			*p* (trend) = 0.45	*p* (trend) = 0.19	
Auxin growth regulator					
0	218 (78.14)	1,005 (78.15)	1.00	1.00	1.00
1	31 (11.11)	154 (11.98)	0.95 (0.61–1.48)	0.66 (0.27–1.63)	1.09 (0.69–1.71)
2–4	24 (8.60)	108 (8.40)	1.13 (0.68–1.89)	0.87 (0.39–1.91)	1.31 (0.66–2.58)
5+	6 (2.15)	19 (1.48)	1.34 (0.49–3.63)	1.69 (0.61–4.73)	–
			*p* (trend) = 0.38	*p* (trend) = 0.51	*p* (trend) = 0.35

^a^ORs adjusted for age and province of residence

^b^ The bold signifies those odds ratios or trends that are statistically significant

## Discussion

This is the first study to examine the risk of HL from exposures to multiple pesticides. Based on the findings of this study, there was a suggestion that the use of multiple insecticides (and more specifically the acetylcholinesterase inhibitors) and fungicides at younger ages (before age 40) might be associated with HL. There was no association with herbicides overall, phenoxy herbicides, or urea herbicides. The risk of HL from reported use of pesticides classified as probably carcinogenic to humans was elevated, but not among those classified as possibly carcinogenic. Somewhat stronger relationships were observed for work-related use than for home-related use of pesticides.

A significant excess of HL associated with use of the insecticide chlorpyrifos had previously been reported from this study, although no individual fungicide was found to be associated with HL [4]. We found an association between use of multiple fungicides and the risk of HL in subjects diagnosed before the age of 40, and a suggestion of an increased risk of HL for all ages with exposure to multiple insecticides. A case–control study in France also found associations between HL and the use of fungicides and several classes of insecticides [6]. On the other hand, Zahm et al. [10] found no association between insecticide use and HL. One reason we may have observed an association with insecticides is because many of the insecticides reportedly used in this study are those classified as probably carcinogenic to humans. The Spearman correlation coefficient between the number of insecticides used and the number of probably carcinogenic pesticides used in the CCSPH was *r* = 0.71 (*p* < 0.0001).

The French case–control study found a large positive association between HL and occupational exposure to urea herbicides (OR 10.8, 95 % CI 2.4–48.1) [6]. However, our study did not find any significant association with this group of herbicides. This may be because the individual urea herbicides reported by the few participants who used these were different in the two studies. However, the especially small number of individuals (5 cases and 4 controls) exposed to urea herbicides in the French case–control study increases the possibility of chance findings.

Previous HL studies have not examined pesticides by their evidence of carcinogenicity, which represents a different approach. The larger odds ratios seen for pesticides classified as probably carcinogenic than for those classified as possibly carcinogenic in this study suggest that this may be a useful method of classifying pesticides to assess their role in the development of HL and other cancers in humans. The increased risk associated with the probably carcinogenic pesticides also shows that pesticides with a high evidence of carcinogenicity, as classified by IARC or EPA, are of greater concern for HL.

This was the first study to examine the risk of HL from pesticide exposure by age groups. Based on the analyses stratified by age, subjects younger than 40 had a greater risk of HL from use of pesticides and fungicides than individuals older than or equal to 40. The increase in risk associated with subjects of younger ages may indicate that there is some underlying factor common to people younger than 40 that puts them at an increased risk of HL. This may be related to the subtype of HL since the distribution of HL subtypes varies by age. For instance, the nodular sclerosis subtype is more commonly seen in young adults and is less frequently observed in the elderly [21]. Thus, analysis by subtype in studies with larger numbers of cases could be informative for future analyses evaluating the risk of HL from pesticide exposure. Another important risk factor for HL is the Epstein–Barr virus (EBV) [3], which causes infectious mononucleosis in adolescents or young adulthood 35–50 % of the time [22]. Studies have shown that approximately 25–50 % of HL cases are EBV-positive [3]. EBV positivity among HL cases tends to be higher among children under 10 years old and in the older age group, but less frequent in young adults [3, 23], where we observed greater risks. Furthermore, EBV positivity is mostly associated with the mixed cellularity subtype [24–27]. The differences in EBV positivity found by age and histologic type may help provide another explanation for the differences in the risk of HL found among older versus younger age groups. Since we lacked information on EBV positivity among individuals, it was not possible to evaluate interaction between EBV, immune system function, pesticides and HL in our study.

In evaluations by mode of action, the risk of HL was associated with using insecticides that act by inhibiting acetylcholinesterase, but not with other types of action. Other HL studies have not examined pesticides in this manner. Acetylcholinesterase inhibiting pesticides are largely comprised of organophosphates and carbamates. There has been little or no evidence that organophosphates or carbamates may be carcinogenic from an experimental point of view [20], but they have been associated with excesses for some lymphatic and hematopoietic cancers in some epidemiologic studies [28]. Another explanation for the significant findings with the acetylcholinesterase group is that it is due to the pesticide chlorpyrifos, which has previously been shown to be associated with an increased risk of HL in this study. To test this, we analyzed HL risk from acetylcholinesterase inhibitors adjusting for chlorpyrifos use (results not presented). Although the OR estimates decreased slightly, the positive associations remained, suggesting that the associations observed with the acetylcholinesterase inhibitors are not entirely due to exposure to chlorpyrifos. Given the magnitude of the association for the acetylcholinesterase group in this study, pesticides that act in this manner deserve further evaluation.

This study also evaluated work- versus home-related pesticide exposure. The stronger relationships observed with work-related pesticide exposure are consistent with the tendency for workers to be exposed to higher quantities and frequencies of use than occurs with residential use. Thus, grouping home use and occupational use together may result in exposure misclassification and lead to a dilution of risk estimates. Recall of pesticide use, however, is generally expected to be more accurate among workers than the general population [29, 30]. This would likely lead to non-differential misclassification among residential users, which would typically bias the OR toward the null.

In our study, 12–15 % of the study population was comprised of proxy respondents or respondents whose status was unknown. We conducted a sensitivity analysis excluding these respondents to assess the effects of any potential exposure misclassification that may be associated with proxy interviews. Methodologic studies have shown that proxy respondents cannot provide as much detail about pesticide use as the subject themselves [31, 32]. Our sensitivity analyses generally demonstrated higher ORs when proxies were excluded for most exposure groups. This suggests that there was non-differential misclassification of pesticide exposure estimates, which may have biased the ORs toward the null when proxies were included [31].

This study has several strengths. It is a population-based study of incident cases. This increases the generalizability of the findings across Canada. The study collected information for a large number of important pesticides and on a variety of possible HL confounders. We also employed several unique methods for classifying pesticides including their carcinogenic potential and mode of pesticidal action to provide alternative assessments of the pesticide–HL relationship. There are some limitations to this study. Information on pesticide use was based on self-reported information provided in the postal and telephone interview questionnaires. Thus, errors in reporting may have occurred. A validation study was conducted of the modified questionnaires used in this study. Results indicated that the concordance between volunteer farmers’ questionnaires and the records of purchases (accessed through their local agrochemical supplier) was excellent [15]. The actual effects of any exposure misclassification would depend upon whether pesticide use was recalled differentially by cases and controls. Non-differential misclassification, where recall errors are similar for cases and controls, would tend to bias the odds ratio estimates toward the null. Differential recall between cases and controls is a concern in case–control studies and could bias ORs in either direction, although the bias toward false positive associations is most often of concern. The potential for differential exposure misclassification has been evaluated in other studies of pesticide use by farmers. Data from the National Cancer Institute studies found little evidence for differential recall of pesticides by farmers [33]. However, our study included farmers and individuals from other occupations. Although pesticide use recall from farmers or pesticide applicators has been validated in some settings, this has not been done for other occupations. Since farmers are heavily involved in all aspects of farm operation, they would have a better memory of past pesticides used through the many tasks they need to perform [31]. There may be a greater likelihood of exposure misclassification from other occupations. This study also examined the risk of HL using a number of different exposure classification methods, raising the issue of multiple testing. This may have led to some positive findings by chance. However, the non-significant associations seen with the herbicides that regulate auxin growth or the stronger associations seen with work versus home exposures are consistent with mechanistic or exposure scenarios for humans.

In conclusion, previous studies have suggested that HL might be associated with exposure to pesticides, but it has generally been less compelling than for other lymphatic and hematopoietic cancers [34]. This study provides additional information on the relationship between HL and pesticide exposure. The findings suggest a potential for an increased risk of HL from exposure to fungicides, insecticides, and those pesticides previously classified as probably carcinogenic to humans. It also suggests that examining pesticides by their mode of action might be a useful analytical technique in future studies. Future studies should also consider age of onset and subtype of HL in the assessment of relationships with pesticide exposure.

## Appendix 1

**List of “probably carcinogenic” and “possibly carcinogenic” pesticides used by subjects in the study**

**Possibly carcinogenic pesticides**

2,4-D

2,4-DB

2,4,5-T

Aldrin

Arsenic

Asulam

Benomyl

Bromoxynil

Carbaryl

Chlordane

Chlorothalonil

Cypermethrin

DDT

Dicamba

Dichlorprop

Diclofop methyl

Dieldrin

Dimethoate

Dinoseb

Diuron

Dual (S-metolachlor)

Ectiban (ambush) (Permethrin)

Formaldehyde

Heptachlor

Kelthane (Dicofol)

Lindane

Linuron

Mancozeb

MCPA

MCPA ester

MCPA-K

MCPB

Mecoprop

Methidathion

Metiram

Paraquat

PCP

Piperonyl butoxide

Propoxur

Quintozene (PCNB)

Rulene

Toxaphene

Triallate

Trichloroacetic acid

Trifluralin

**Probably carcinogenic pesticides**

Aldrin

Arsenic

Carbaryl

Chlordane

Chlorothalonil

DDT

Diclofop methyl

Dieldrin

Diuron

Ectiban

Formaldehyde

Heptachlor

Mancozeb

PCP

Propoxur

Rulene

Toxaphene

## Appendix 2

**List of pesticides used by subjects belonging to each of the modes of action**

**Acetylcholinesterase inhibitors**

Aldicarb

Azinphosmethyl

Carbaryl

Carbofuran

Chlorpyrifos

Coral

Diazinon

Dimethoate

Fenthion

Malathion

Metamidophos

Methidathion

Methomyl

Metsystox

Propoxur

Trichlorfon

Triumph

**Sodium channel Activators**

Cypermethrin

DDT

Decis

Ectiban

**GABA receptors-gated chloride channel inhibitors**

Aldrin

Chlordane

Cypermethrin

Decis

Dieldrin

Endrin

Heptachlor

Toxaphene

**Auxin Growth Regulators**

2,4,5-T

2,4-D

2,4-DB

Acclaim

Dicamba

Dichlorprop

Diclofop methyl

Fenoprop

Lontrel

MCPA

MCPA ester

MCPA-K

MCPB

Mecoprop

Picloram
